# miRNA-29c Suppresses Lung Cancer Cell Adhesion to Extracellular Matrix and Metastasis by Targeting Integrin β1 and Matrix Metalloproteinase2 (MMP2)

**DOI:** 10.1371/journal.pone.0070192

**Published:** 2013-08-06

**Authors:** Heyong Wang, Yingchao Zhu, Mingchuan Zhao, Chunlian Wu, Peng Zhang, Liang Tang, Huijun Zhang, Xiaofeng Chen, Yaoqin Yang, Gentao Liu

**Affiliations:** 1 The Central Laboratory, Shanghai Pulmonary Hospital, Tongji University School of Medicine, Shanghai, China; 2 Institute of Oncology, Tongji University School of Medicine, Shanghai, China; 3 Center for Translational Medicine, Shanghai Pulmonary Hospital, Tongji University School of Medicine, Shanghai, China; 4 School of Life Science, China West Normal University, Nanchong, Sichuan Province, China; Lerner Research Institute, United States of America

## Abstract

Our pilot study using miRNA arrays found that miRNA-29c (miR-29c) is differentially expressed in the paired low-metastatic lung cancer cell line 95C compared to the high-metastatic lung cancer cell line 95D. Bioinformatics analysis shows that integrin β1 and matrix metalloproteinase 2 (MMP2) could be important target genes of miR-29c. Therefore, we hypothesized that miR-29c suppresses lung cancer cell adhesion to extracellular matrix (ECM) and metastasis by targeting integrin β1 and MMP2. The gain-of-function studies that raised miR-29c expression in 95D cells by using its mimics showed reductions in cell proliferation, adhesion to ECM, invasion and migration. In contrasts, loss-of-function studies that reduced miR-29c by using its inhibitor in 95C cells promoted proliferation, adhesion to ECM, invasion and migration. Furthermore, the dual-luciferase reporter assay demonstrated that miR-29c inhibited the expression of the luciferase gene containing the 3′-UTRs of integrin β1 and MMP2 mRNA. Western blotting indicated that miR-29c downregulated the expression of integrin β1 and MMP2 at the protein level. Gelatin zymography analysis further confirmed that miR-29c decreased MMP2 enzyme activity. Nude mice with xenograft models of lung cancer cells confirmed that miR-29c inhibited lung cancer metastasis in vivo, including bone and liver metastasis. Taken together, our results demonstrate that miR-29c serves as a tumor metastasis suppressor, which suppresses lung cancer cell adhesion to ECM and metastasis by directly inhibiting integrin β1 and MMP2 expression and by further reducing MMP2 enzyme activity. The results show that miR-29c may be a novel therapeutic candidate target to slow lung cancer metastasis.

## Introduction

Today, lung cancer is one of the most common cancers. More than 90% of lung cancer patients die of metastasis rather than from their primary tumors, suggesting that metastasis is a key prognostic factor [1], [2]. Tumor progression and metastasis is a complex process involving many different biological players. One of the critical regulators that involved in this process is a microRNA (miRNA) [3].

Mature miRNAs are short, single-stranded, endogenous and non-coding RNAs consisting of about 22 nucleotides, which regulate genes at the post-transcriptional level during the translation process. They can target the 3′-UTR (untranslated regions) of mRNA, which functionally leads to a translational inhibition or deregulation of the target mRNA [4]. miRNAs have a major influence on various biological processes, including cell differentiation, proliferation, apoptosis, stress resistance, fat metabolism, and development. Therefore, they play a crucial role in different diseases including cancer [3]. Moreover researches indicate that some miRNAs can function as oncogenes or tumour suppressors. MiRNAs play an important role in tumorigenesis and tumor progression, including proliferation and metastasis [3], [5]–[8]. For example, miR-10b promotes breast tumor metastasis, while miR-335 and miR-126 suppress the metastasis [9], [10]. Therefore, the next generation of therapeutic targets for malignant tumors may be miRNAs [11].

The miR-29 family is a conserved family of miRNAs including miR-29a, miR-29b, miR-29c, and miR-29d. Recently, the expression levels of many miR-29 family members were found to be reduced in a variety of cancers. For example, Sengupta and his colleagues have shown that miR-29c is down-regulated in nasopharyngeal carcinomas [12], while Fabbri and his colleagues discovered that the miR-29 family, including miR-29c, targets DNMT3A and DNMT3B in lung cancer tissues and cells [13].

In the present study, Our earlier pilot study found a nearly fourfold differential expression of miR-29c between high-(95D) and low-metastatic (95C) cancer cell lines (Details in Table S1). Several studies have taken advantage of this difference between the twin cell lines in relation to invasion and metastasis [14], [15]. However, the role of miR-29c in lung cancer has yet to be thoroughly explored and most of its overall biological function remains unknown. Here, we show evidence that miR-29c functions as a metastasis suppressor that inhibits lung cancer cell adhesion to ECM and migration *in vitro*, and suppresses cancer cell distant metastasis *in vivo*. We further confirmed that miR-29c can directly downregulate integrin β1 and MMP2 expression by targeting the 3 ′-untranslation sequence, inhibit the protein levels of integrin β1 and MMP2, and reduce MMP2 enzyme activity. The results show that miR-29c may be a novel therapeutic candidate target or strategy for seeking to control lung cancer metastasis.

## Materials and Methods

### Ethics

All animal experiments were performed using female nude mice (6–7 week old). The mice were purchased from the SLAC Laboratory Animal Ltd., Co. (Shanghai, China) and cared in accordance with the National Institutes of Health Guide for the Care and Use of Laboratory Animals. All animal experimental protocol was approved by Institutional Animal Care and Use Committee of Tongji University (IACUC No. 1201).

### Cell lines and cell culture

The paired low-metastatic 95C and high-metastatic 95D cell lines were subcloned from a low differentiated human large cell lung carcinoma cell line PLA-801. The cells were well authenticated and published by several research groups [14], [15]. The cells were kindly provided by Professor Ying-Lin Lu, Department of Pathobiology, Institute of Basic Medical Sciences, Academy of Military Medical Sciences, Beijing, China on Dec. 5,2009. As described The 95C and 95D cells were cultured in RPMI 1640 (Invitrogen, USA) with 100 units/mL penicillin, 100 μg/mL streptomycin and 10% calf bovine serum, and grown at 37°C in atmosphere with 5% CO_2_. Cells were used for functional studies within 6 months after thawn from liquid nitrogen tank.

### MicroRNAs array and qRT-PCR analysis

After performing a simple migration assay to screen high- and low-metastatic cells, we conducted a transwell assay as an index of Matrigel invasion *in vivo* using non-invasive 95C cells in the upper chamber of the non-coated transwell insert (24-well insert; pore size 8 μm; Corning, USA) and 95D cells in the bottom using a Matrigel (50 μg/ml) coated transwell insert to amplify the differential expression of miRNAs. Then miRNAs differential expression between 95C and 95D was measured using a miR human_01_H10.1_080277 miRNA array (LC Sciences Houston, USA). All data was deposited at Gene Expression Omnibus (GEO). The accession number is GSE47788. The measured miRNAs with a statistical difference (*p*<0.01) were considered to be significantly differentially expressed.

Quantitative real-time PCR (qRT-PCR) was conducted to measure the expression levels of 95C and 95D cells. Total RNA from wild type 95C and 95D were isolated using Trizol reagent (Invitrogen, USA) according to the manufacturer′s instruction. The total RNA (50 ng) was reverse-transcribed with miR-29c stem-loop RT primers or U6 RT primers (Shanghai Extended Nature Biotech Co., Ltd) by using a RevertAid™ First Strand cDNA Synthesis Kit (Fermentas, USA) according to the manufacturer's protocol to form cDNA. Real-time PCR was performed using a SYBR® Premix Ex Taq™ Kit (TaKaRa, Japan) with miR-29c or U6 PCR primers (Shanghai Extended Nature Biotech Co., Ltd). The reactions were performed using a ABI7500 Real-Time PCR System (Applied Biosystems). All data for each sample were collected in triplicate and assessed using 2^−ΔΔCt^ relative quantitative analysis.

### Gain-of-function and loss-of-function for miR-29c

The gain-of-function assay by miR-29c mimics and the loss-of-function assay by miR-29c inhibitor *in vitro* were carried out by cell transfection using the method mentioned below. Oligonucleotide sequences of miR-29c mimics, inhibitor and their negative control are:

miR-29c mimics (29c):

Sense: 5′-UAGCACCAUUUGAAAUCGGUUA-3′,

Anti-sense: 5′-UAACCGAUUUCAAAUGGUGCUA-3′;

miR-29c mimics negative control(MiNC):

Sense: 5′-UCACAACCUCCUAGAAAGAGUAGA-3′,

Anti-sense: 5′-UCUACUCUUUCUAGGAGGUUGUGA-3′;

miR-29c inhibitor(29ci): 5′-UAACCGAUUUCAAAUGGUGCUA-3′

miR-29c inhibitor negative control(IhNC): 5′- UCUACUCUUUCUAGGAGGUUGUGA-3′.

All these oligonucleotides were chemosynthesized from Extended Nature Biotech, Shanghai.

### Cell transfection

The 95C or 95D cells were cultured to about 80% confluence in 6-well plates and were transfected with Lipofectamine 2000 (Invitrogen, USA) according to the manufacturer's instructions. Cells in each well of a 6-well plate were transfected with 100 nM mimics or 200 nM inhibitor. 24–48 hours after transfection, the cells were harvested for further experiments, including proliferation, adhesion, migration, and invasion.

### Cell proliferation assays

The proliferation of the transfected cells were evaluated by MTT assay. About 2×103 cells were seeded in a 96-well plate with 100 μl medium in each well. After 24 h, 48 h 72 h and 96 h, 20 μl MTT (5 mg/ml) was added in a 96-well plate and incubated for 4 h before reading at 530 nm in a plate reader. All independent experiments were run in triplicate.

### Cell adhesion to extracellular matrix(ECM) assays

The transfected cell adhesion to Matrigel were evaluated by MTT assay and counting. In the MTT assay, 96-well plates were pre-coated with 40 μl of 50 μg/ml Matrigel (BD Biosciences, USA) and then 1×104 cells were plated into every well in a 96-well plate with 100 μl medium and the cells were incubated at 37°C for 2 h. Next we performed the MTT assay previously described after thrice washing away the non-adherent cells with PBS. In the counting assay, the 95D and 95C cells were transfected with lentivirus of NC-CMV-GFP-LV (Shanghai Genechem) to stably express the GFP gene. The 95D-GFP and 95C-GFP cells were transfected with 100 nM mimics or 200 nM inhibitor, respectively, trypsinized, washed twice in 1× PBS, and then 1×104 cells with100 μl medium were plated into every well in a 96-well plate pre-coated with 40 μl of 50 μg/ml Matrigel and the cells were incubated at 37°C for 30 min.The non-adherent cells were washed out 3 times with PBS, and the number of cells that adhere Matrigel to were counted under an inverted microscope(Olympus Corp. Tokyo, Japan) at 100×magnification from 3 randomly selected green fields in each well. The independent experiments were run in three times.

### Cell migration and invasion assays

The potential for migration and invasion of transfected cells were evaluated by a transwell assay. In the migration assay, 2.5×104 cells were cultured in 200 μl medium with 1% calf bovine serum in the upper chamber of a non-coated transwell insert. In the lower chamber, 600 μl medium with 10% calf bovine serum was used as a chemo-attractant to encourage cell migration. In the invasion assay, the upper chamber of the transwell inserts were coated with 50 μl of 2.0 mg/ml Matrigel, and 5×104 cells were plated in the upper chamber of the Matrigel-coated transwell insert. Cells of both assays were incubated for 24 h and cells that did not migrate or invade were removed using a cotton swab. All cells were stained using crystal violet staining and counted under an inverted microscope. We selected four random views to count the cells and the independent experiments were repeated three times.

### Determination of miR-29c targeting sequences by computational prediction

Computational prediction has already been proven to be an effective and efficient method for predicting miRNA's targets. We used TargetScan, PicTar and MiRanda to predict the miR-29c targeting sequences. Targets which were predicted by the three databases and were related to tumor invasion and metastasis were considered relevant to our research.

### Dual-luciferase assay to determine the effects of the target sequences of the 3′-UTR region of integrin β1 and MMP2

The 3′-UTR fragment of the integrin β1 and MMP2 genes, including the miR-29c binding site, was amplified by PCR from 95D cell genomic DNA using the following primers. PCR primers for integrin β1 were:

Intβ1-*Sal*I-UTR1, 5′-TGGAGCTCTAACTGCCCGTGCAAATCCCACAAC-3′ (forward),

Intβ1- *Mlu*I–UTR2, 5′-TGACGCGTAACTTCAGTAAATAGCACTGTA-3′ (reverse).

PCR primers for MMP2 were:

MMP2-*Mlu*I-UTR1, 5′-TGACGCGTAACTGCCTTCGATACACCGGGCCTG-3′ (forward), MMP2-*Hind*III-UTR2, 5′-TGAAGCTTGCACATAGAAAGCACTCTATTAATTC-3′ (reverse).

The integrin β1 and MMP2 gene mutant 3′-UTR fragment containing the miR-29c binding site was amplified from the 3′-UTR fragment using the following primers for integrin β1 by Intβ1-*Sal*I-UTR1+Intβ1-*Mlu*I-*mut*-UTR3(5′-TGACGCGTGAAGAGGTGACAGAAACTAAGT GACATTAAAC-3′) and for MMP2 by MMP2-*Mlu*I-UTR1 + MMP2-*Hind* III*-mut-*UTR (5′-TGAAGCTTGAAGAGCCTGAAGTGTGGCAGCGTCTGGGC-3′) (Underlined bases show the mutation site).

The amplified fragment was cloned into the pMIR-REPORT Luciferase vector (Ambion, USA) at the *Sal*I+ *Mlu* I *and Mlu* I+ *Hind* III site for integrin β1 and MMP2, respectively.

The 95C or 95D cells were co-transfected in 24-well plates containing 200 ng firefly Luciferase vector, 40 ng *Renilla* luciferase pRL-TK vector (Promega, USA) and 100 nM mimics or 200 nM inhibitor. Firefly luciferase acted as a reporter gene and *Renilla* luciferase as a normalized control. The transfected cells were incubated for 48 h. Luciferase activity was measured using the Dual-Luciferase Reporter Assay System (Promega, USA).

### Western blotting

For isolation of total proteins, treated and control cells were washed with 1× PBS, lysed with RIPA buffer (50 m mol /L Tris-base, 150 m mol /L NaCl, 0.1% SDS, 1% Triton X-100, 0.5% sodium deoxycholate, 1 m mol /L sodium orthovanadate, 10 m mol /L sodium fluoride, 1% protease inhibitor cocktail) for 15–20 min on ice. After centrifugation at 12000 rpm for 15 min, the supernatants were collected. and protein concentration was quantified by the BCA protein assay. 20–30 μg of total proteins were dissolved in SDS-PAGE loading buffer, heated at 100°C for 5 min , separated on 10% polyacrylamide gel, transferred to nitrocellulose membranes (Amersham Biosciences). The membranes were blocked in 5% non-fat milk in TBST buffer (Tris Buffer Saline containing 0.1% Tween-20) for 1 h at room temperature, and subsequently incubated overnight at 4°C by the appropriately diluted primary antibodies for rabbit monoclonal anti-human integrin β1 and MMP2 (diluted 1∶1000; Cell Signaling Technology). After extensive washing with TBST buffer, the blots were then incubated with HRP-conjugated secondary antibody for 1 h at room temperature. After extensive washing with TBST buffer, target proteins were detected by enhanced chemiluminescence reagents ECL.

### Gelatin zymography of MMP2 enzyme activity

This experiment sought to detect specific MMP2 and MMP9 enzyme activity. The media without the serum collected from the transfected 95C and 95D cells after 24 hrs were analyzed using a gelatin zymography assay kit (Applygen, China). MMP activity was measured by SDS-PAGE under non-reducing conditions. The gel contained 1% gelatin and 30% acrylamide. Electrophoresis was carried out at 4°C. After washing with the buffer A (containing 2% Triton X-100) from the kit, the gel was incubated in 37°C with the buffer B (containing the necessary metal ion: 5 mmol/CaCl_2_, 1 μmol/L ZnCl_2_). MMP activity was visualized by staining with Coommasie Blue R-250.

### In vivo metastasis assays in nude mice xenograft model

The 95D and 95C cells were transfected with lentivirus of U6-RNAi-(Ubi)-luc-LV (Shanghai Genechem) to stably express the firefly luciferase in lung cancer cells. The 95D-luc and 95C-luc cells were transfected with 100 nM mimics or 200 nM inhibitor, respectively, trypsinized, washed twice in 1× PBS, and resuspended at a concentration of 1–5×10^6^ cells/ml in cold 1× PBS. Female nude mice (6–7 weeks old) were anesthetized by intraperitoneal injection of ketamine (90–110 mg/kg) and xylazine (10 mg/kg) before the intracardiac injections and were placed in the supine position. With a 25-gauge needle, 2–3×10^5^ cells were injected into the left ventricle (volume 0.1 ml) after visualization of arterial blood flow into the syringe. After injection, the mice were placed on heating cages to recover from anesthesia. The cell-injected animals were housed under sterile conditions at Experimental Animal Facility of Tongji University (Shanghai, P.R. China).

For the *in vivo* metastasis assay, lung cancer metastasis was monitored in the living animals by bioluminescent imaging (BLI) using a LB 983 in-vivo imaging system(Berthold) starting 3–4 weeks after implantation. Fifteen minutes before *in vivo* BLI, animals were anaesthetized with 1–3% isoflurane and injected intraperitoneally with D-luciferin (150 mg/ kg in 1× PBS). The experiments were performed using 5 or 6 mice per group and repeated thrice. The development of bone metastases was monitored by X-ray radiography for 6–8 weeks after injection. The mice were anesthetized, placed on a transparent board in prone and lateral positions, exposed to X-rays at 30 kV and 0.5 mA for 10 s in a Fixitron MX-20 Radiography System at the Institute of Traumatology & Orthopedics, Shanghai Academy of Traditional Chinese Medicine.

### Statistical analysis

Quantitative values were presented as mean ± SEM. The Student's *t* and **χ^2^** tests were conducted to compare the corresponding data. Differences with *P*<0.05 were considered to be statistically significant.

## Results

### miR-29c is down-regulated in high-metastatic lung cancer cells

**Figure 1:**
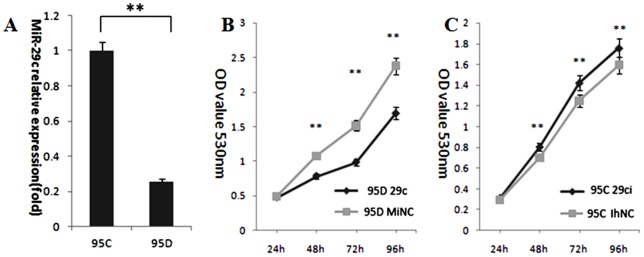
Differential expression of miR-29c in the paired high- and low-metastatic lung cancer cell lines, and the effects of miR-29c on lung cancer cell proliferation *in vitro*. (A) qRT-PCR of miR-29c in 95D and 95C cell lines. Change was calculated using 2^−ΔΔCt^ relative quantitative analysis; **p<0.01 (Student's *t*-test). Experiments were repeated at least thrice (n = 3). (B) miR-29c inhibits cellular proliferation in 95D cells by MTT assay. **p<0.01(Student's *t*-test, n = 3). (C) miR-29c inhibitor increased cellular proliferation in 95C cells by MTT assay. **p<0.01 (Student's *t*-test, n = 3).

In this study, the 95C and 95D cells lines were selected because they originated from the same individual and there is a dramatic difference in their metastatic behavior. To identify the differential expression of miRNAs between 95C and 95D cells. First, a primary screening was conducted using a simple Matrigel invasion transwell assay before miRNA array. We found a significant differential expression of miRNAs between 95C and 95D cells using miRNA array (Details in Table S1). To study the possible role of miRNAs that inhibit lung cancer cell metastasis and potential candidates target genes, we focused on miR-29c because it is down-regulated in high-metastatic 95D cells and up-regulated in low-metastatic 95C cells. The finding may imply miR-29c function as a tumor metastasis suppressor. The data from qRT-PCR further confirmed the different expression levels of miR-29c between 95C and 95D cells. This result is consistent with the miRNA array results. (miR-29c showed a 3.8 fold times higher in 95C than 95D from Figure 1A).

### miR-29c suppresses cell proliferation in 95D cells

To determine whether miR-29c suppresses lung cancer cell proliferation, miR-29c mimics (miR-29c) and mimics negative control (MiNC) were transfected into 95D cells, while miR-29c inhibitor (29ci) and inhibitor negative control (IhNC) were transfected into 95C cells. MTT assays were conducted to measure cell proliferation. Results (Figure 1B) suggest that miR-29c has an inhibitory effect on the proliferation of 95D cells. There was no difference at 24 h in the proliferation of 95D cells between the miR-29c and MiNC groups (*p*>0.05). However, there was a significant difference between the two groups after 48 h, 72 h and 96 h of incubation (*p*<0.01). The inhibitory efficiencies were 37.5%, 54.4% and 40.3% (Figure. 1B), respectively. Therefore, miR-29c's inhibition of 95D cell proliferation is a time-dependent process.

In 95C cells transfected with miR-29c inhibitor (miR-29ci), proliferation increased in a time dependent manner. The proliferation rates between the miR-29ci and IhNC groups did not vary (*p*>0.05) at the 24 h point of incubation. However, there was a statistically significant between the two groups after 48 h, 72 h and 96 h of incubation with proliferation rates of 15.2%, 13.7% and 10.4% (Figure 1C, *p*<0.01), respectively. Therefore, miR-29c promoted 95C cell proliferation in a time-dependent manner.

### miR-29c inhibits cell adhesion to ECM in vitro

**Figure 2:**
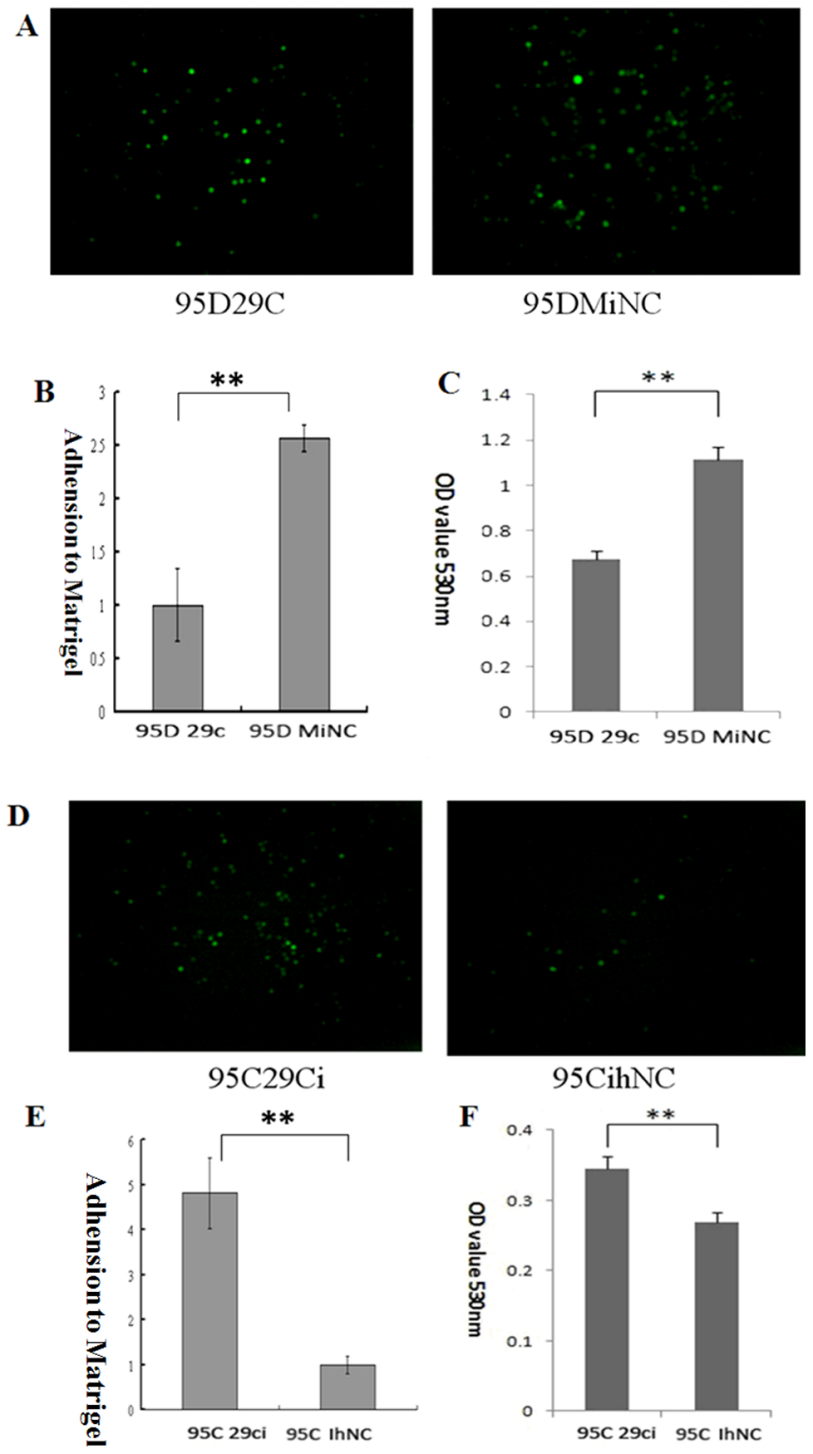
Effect of miR-29c on lung cancer cell adhesion to ECM *in vitro*. (A) 95D cells transfected with miR-29c mimics adhesion to Matrigel was measured by counting as described in Materials and Methods. Representative fields of 95D 29c and 95D MiNC (magnification ×100). (B) Average adhesion cell number per random field. **P<0.01(Student's *t*-test, n = 3). (C) miR-29c inhibits 95D cell adhesion (MTT assay). **p<0.01(Student's *t*-test, n = 3). (D) Representative fields from 95D 29ci and 95C IhNC lung cancer cells (magnification ×100). (E) Average adhesion cell number per random field. **P<0.01(Student's *t*-test, n = 3). (F) Suppression of miR-29c enhanced cell adhesion in 95C cells (MTT assay). **p<0.01 (Student's *t*-test, n = 3).

Adhesion to ECM is a key step when cancer cells plant themselves in distant locations during metastasis. Matrigel containing the basement membrane components can simulate cell adhesion *in vitro.* We took advantage of this property and performed an adhesion assay. In 95D cells transfected with miR-29c mimics (miR-29c) and mimics negative control (MiNC), the number of cells that adhere to the Matrigel decreased significantly (*p*<0.01, Figure 2 A–C) after miR-29c transfection compared to those transfected with MiNC. However, in 95C cells transfected with miR-29c inhibitor (29ci) and inhibitor negative control (IhNC), miR-29c inhibitor has significantly increased 95C adhesion to matrigel when cells transfected with miR-29c inhibitor compared to those transfected with IhNC (*p*<0.01, Figure 2 D–F). These results indicate that mir-29c can decrease the number of adhesion cells in high-metastasis 95D cells, while the inhibition of mir-29c can increased cell adhesion in low-metastatic 95C cells (Figure 2).

### miR-29c inhibits cell invasion and migration in vitro

**Figure 3:**
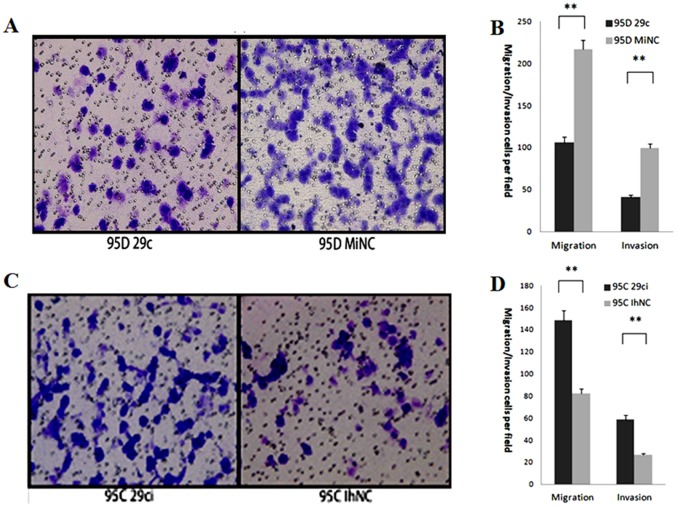
Effect of miR-29c on lung cancer cell migration and invasion *in vitro*. (A) In a Matrigel invasion assay, miR-29c mimics transfected 95D cells vs MiNC transfected cells in a 200× light scope after crystal violet staining. (B) Matrigel invasion and transwell migration: 95D cells were counted in a light scope in four random views. **p<0.01 (Student's *t*-test, n = 4 ). (C) In a Matrigel invasion assay, miR-29c inhibitor transfected 95C cells vs IhNC transfected cells in a 200× light scope after crystal violet staining. (D) Matrigel invasion and transwell migration: 95C cells were counted in a light scope in four random views. **p<0.01 (Student's *t*-test, n = 4).

Transwell assay is widely used to measure cellular behavior during migration and invasion, key steps in tumor metastasis. We set a Matrigel membrane or the transwell itself with a chemo-attractant to simulate cell invasion and migration behavior *in vitro*. In 95D cells after miR-29c and MiNC transfection, a visible significant difference was seen with fewer miR-29c transfected cells counted than MiNC transfected cells (cell numbers reduced 104.2% in migration assay and 136% in invasion assay, *p*<0.01). This finding may indicate that upregulation of miR-29c inhibits cell invasion and migration(Figure 3A and B). In contrast, transfection of 95C cells with miR-29c inhibitor and IhNC showed an opposite result; more miR-29c inhibitor transfected cells passed through the matrigel and the transwell membrane (cell numbers increased about 79.7% in the migration assay and 97.4% in the invasion assay, *p*<0.01) compared to the 95C cells transefected with IhNC (Figure 3 C and D). These data show that miR-29c influenced cell invasion and migration behavior *in vitro*.

### miR-29c directly targets 3′-UTR of the integrin β1 and MMP2 gene

**Figure 4:**
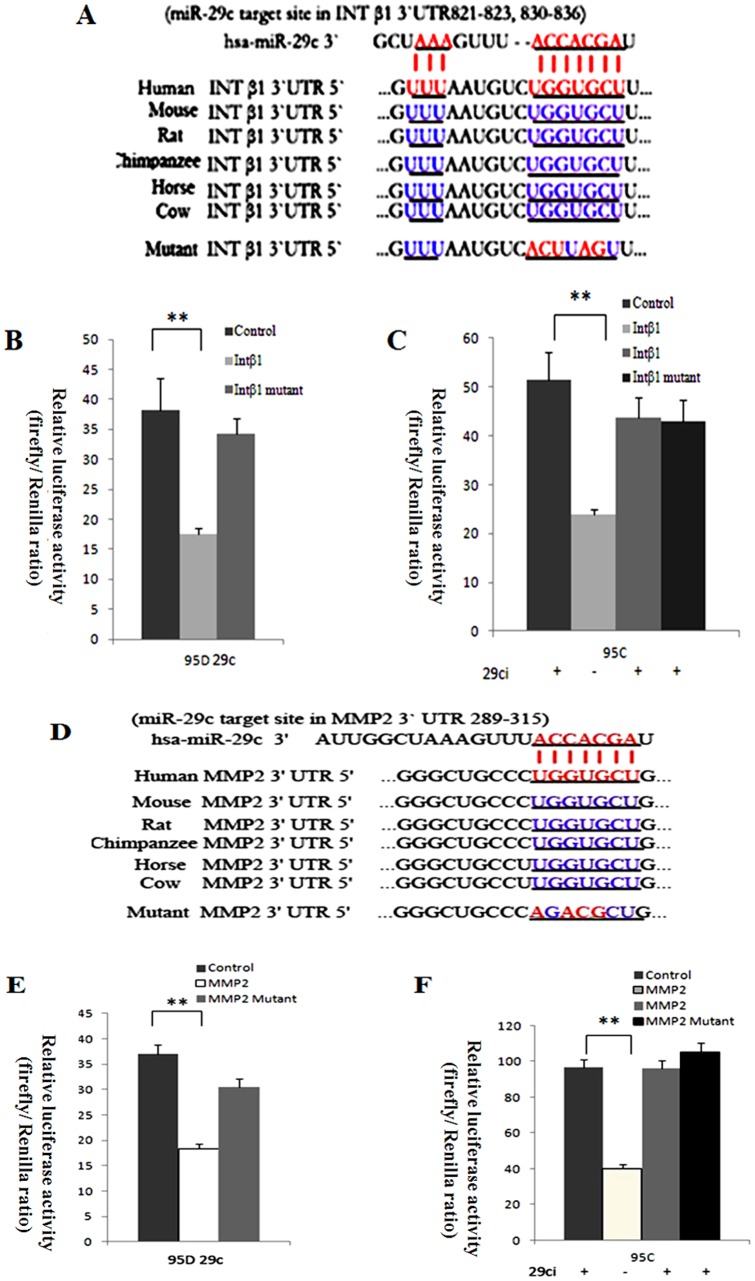
MiR-29c directly targets 3′-UTRs of the human integrin β1 (Intβ1) and MMP2 gene. (A) Conserved targeting of 3′-UTRs of the human int β1 by miR-29c (underlined). The wild-type and mutant sequences of int β1 3′-UTR are listed for comparison. (B) Dual-luciferase reporter assay for target gene int β1 in 95D cells transfected with miR-29c mimics. **p<0.01 (Student's *t*-test, n = 3 ). (C) Dual-luciferase reporter assay for target gene int β1 in 95C cells with or without transfecting mir-29c inhibitors. **p<0.01 (Student's *t*-test, n = 3). (D) miR-29c binding sites in the MMP2 3′UTR region (underlined); binding site is highly conserved in vertebrate animals, including humans. The wild-type and mutant sequences of MMP2 3′UTR are listed for comparison. (E) Dual-luciferase reporter assay of MMP2 mRNA in 95D cells transfected with miR-29c mimics. **p<0.01 (Student's *t*-test, n = 3). (F) Dual-luciferase reporter assay of MMP2 mRNA in 95C cells with or without transfecting mir-29c inhibitors. **p<0.01 (Student's *t*-test, n = 3).

To further explore the mechanisms by which miR-29c suppresses lung cancer cell invasion and metastasis, we analyzed probable downstream tumor metastasis-related genes. Computational prediction was used to find out the most likely target gene. We took advantage of the TargetScan, PicTar and MiRanda databases of predicted microRNA targets to analyze taregets of miR-29c. The 3′-UTRs of integrin β1 (Intβ1) and MMP2 bear miR-29c-binding sites. 3′-UTR of integrin β1 and MMP 2 of miR-29c-binding sites has highly conservative sequences in mammals (Figure 4A and D). The studies into the putative functions of miR-29c in cellular adhesion and invasion encouraged us to further research into integrin β1 and MMP2.

To determine whether integrin β1 and MMP2 are suppressed by miR-29c through binding to their mRNA 3′-UTR, we performed a dual-luciferase assay to verify the relationship between the two target gene and miR-29c. The wild-type and mutant integrin β1 and MMP 2 mRNA 3′-UTR were inserted into the downstream region of a luciferase reporter gene from pMIR-REPORT vector, and namely the wild-type integrin β1 3′-UTR vector, MMP 2 3′-UTR vector, mutant integrin β1 3′-UTR vector and mutant MMP2 3′-UTR vector, respectively. We used the pMIR-REPORT Luciferase vector as a control because it would not be influenced by any miR-29c mimics or inhibitor, and can also act as a standard control for transfection efficiency. Therefore, *Renilla* luciferase vector (pRL-TK vector) and firefly luciferase vector were co-transfected with mimics or inhibitor. In 95D cells with miR-29c mimics, the luciferase activity of wild-type integrin β1 and MMP2 vector relative ratio was inhibited about 2.00 times compared to the control (Figure 4 B and C, *p*<0.01). Though the mutant integrin β1 and MMP2 vector in 95D cells showed a lower luciferase activity than the control, there was no significant difference (*p*>0.05). In 95C cells, inhibition of miR-29c has increase in integrin β1 and MMP2 luciferase activity (Figure 4 E and F *p*<0.01) compared to cells that were not stimulated with miR-29c inhibitor due to the integrin β1 and MMP2 vector luciferase activity, which was inhibited by endogenous miR-29c. Similarly, mutant integrin β1 and MMP2 in 95C cells showed no significant reaction to the control (*p*>0.05). Apparently, miR-29c targets integrin β1 and MMP2 directly since gain-of-function of miR-29c in 95D cells significantly decreased the integrin β1 and MMP2 vector luciferase activity, and loss-of-function of miR-29c inhibitor in 95C cells showed that integrin β1 and MMP2 retains its expression level only when the cells are not exposed to miR-29c inhibitor (Figure 4).

### miR-29c inhibits endogenous protein expression of the integrin β1 and MMP2

**Figure 5:**
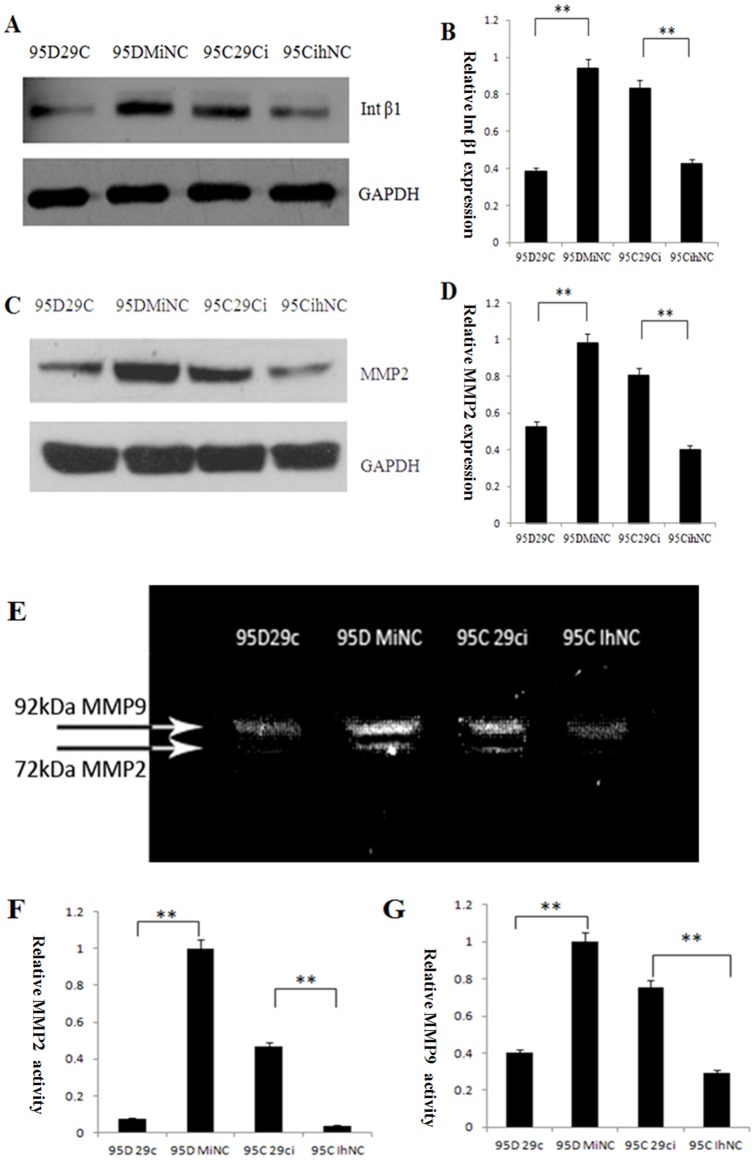
miR-29c inhibits endogenous integrin β1 (Int β1) and MMP2 protein expression and decreases MMP2 enzyme activity in lung cancer cells. (A) Effects of miR-29c on endogenous integrin β1 protein. Changes in protein expression of Int β1 was determined by Western blotting analysis in 95D 29c , 95D MiNC, 95C 29ci and 95C IhNC. GAPDH was used as an internal loading control. (B) The gray levels of Western blotting are shown by bar gragh. miR-29c mimics inhibit endogenous integrin β1 in 95D cell lines (95D 29c and 95D MiNC), while miR-29c inhibitors promote integrin β1 protein expression. **p<0.01 (Student's *t*-test, n = 3). (C) Effects of miR-29c on endogenous MMP2 protein levels by Western blotting. (D) The gray levels of Western blotting are shown by bar gragh. **p<0.01 (Student's *t*-test, n = 3). (E) Effects of miR-29c on MMP2 and MMP9 enzyme activity in lung cancer by gelatin zymography. (F) The gray level of every band was measured to check the differences in MMP2 enzyme activity in 95C and 95D cells (The brightest band [95D MiNC] was set to 1 unit to compare with other gray levels). **p<0.01 (Student's *t*-test, n = 3). (G) The gray level of every band was measured to check the difference in MMP9 enzyme activity between 95C and 95D cells (The brightest band [95D MiNC] was set to 1 unit to compare with other gray levels). **p<0.01 (Student's *t*-test, n = 3).

To further investigated the effects of miR-29c on integrin β1 and MMP2 protein expression by Western blotting. In 95D cells transfected with miR-29c mimics, the expression of integrin β1 and MMP2 at the protein levels were significantly less than that of the negative control of cells transfected with MiNC (Figure 5, *p*<0.01). In 95C cells, compared to cell transfected with IhNC, Western blotting results indicated integrin β1 and MMP2 preotein expression were upregulated after transfected with miR-29c inhibitor in 95C(Figure 5, *p*<0.01). These data show integrin β1 and MMP2 are direct targets of miR-29.

### miR-29c inhibits endogenous MMP2 enzyme activity

Since MMP activity is required when cancer cells, including lung cancer, migrate and invade adjacent tissue, cells and extracellular matrices, we further investigated whether down-regulation of MMP2 transcription by miR-29c effects on MMP2 enzyme activity using gelatin zymography. Gelatin zymography assay was conducted based on the basic theory that MMP2 degrades gelatin. MMP2 in the collected media from 95D cells transfected with miR-29c mimics showed lower enzyme activity than the MiNC transfected sample, as shown by the bands at 72 kDa, which represents MMP2 activity (Figure 5E and F). At the same time, gelatin zymography also showed that the intensity of the bands at 92 kDa, which represents MMP9 activity, was reduced more in 95D cells transfected with miR-29c mimics than the MiNC transfected sample. In contrast, in the 95C cells the band representing MMP2 caused by the inhibition of 95C cells transfected with miR-29c also showed a higher protein enzyme activity for MMP2 and MMP9 than that of cells transfected with IhNC (Figure 5 E and G). These results indicate that miR-29c directly targets MMP2 at transcription level eventually to lower MMP2 enzyme activity.

### miR-29c inhibits tumor invasion and metastasis in a nude mouse xenograft model

**Figure 6:**
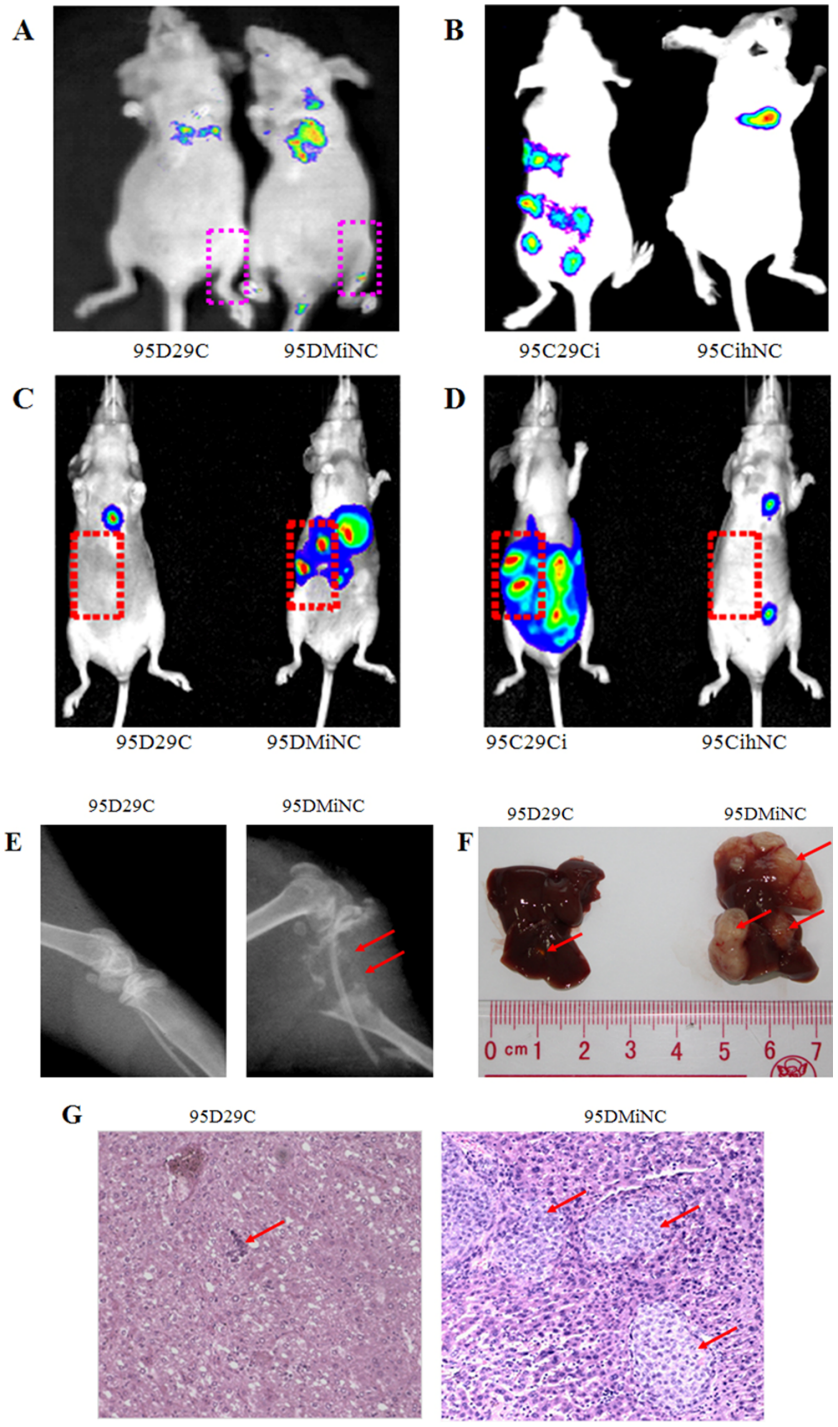
MiR-29c inhibits tumor metastasis *in vivo*. (A) miR-29c mimics suppress 95D cells metastasis to bone *in vivo* as shown by bioluminescence imaging after injection of 95D cells transfected with MiNC for 3–4 weeks, (□bone metastasis sites). (B) miR-29c inhibitor promotes 95C cells metastasis *in vivo* as shown by bioluminescence imaging. (C) miR-29c mimics can suppress 95D cells metastasis to liver *in vivo* as shown by bioluminescence imaging (□liver metastasis sites). 95D MiNC cells can metastasize to bone. (D) miR-29c inhibitor promotes 95C cells metastasis to liver *in vivo* as shown by bioluminescence imaging. (E) miR-29c mimics suppress 95D cells metastasis to bone *in vivo.* Bone metastasis shown by X-ray radiography 6–8 weeks after injection. The bone was destroyed by osteolytic metastasis (red arrow) caused by 95D cells transfected with MiNC. (F) miR-29c mimics suppress 95D cells metastasis to liver *in vivo.* Representative anatomical photos of livers from mice injected with 95D 29c or 95D MiNC cells. Liver metastasis was not found 6–8 weeks after injection from 95D 29c (red arrow). (G) Representative liver tissue sections from each group were shown (hematoxylin and eosin stain; magnification, ×100). Red arrows indicate liver metastasis.

We measured the metastasis suppressing potential of miR-29c *in vivo* in a widely used nude mouse metastasis xenograft model. For 95D cells transfected with MiNC, the tumor grew and spread rapidly including into bone, liver, brain, tail and eyes, which was visible using a bioluminescence imaging system. X-ray radiography showed that the thighbone exhibited serious osteolysis. After transfection with miR-29c mimics, the metastasis rate was significantly reduced (Figure 6 A, C, E, F and G). On the other hand, 95C cells transfected with IhNC showed less metastasis than 95C cells when miR-29c inhibitor was transfected. We have found that the number of metastatic nodes dramatically increased in the nude mice injected with 95C cells transfected with miR-29c inhibitor compared to those transfected with IhNC (Figure 6 B and C). The findings showed that inhibition of miR-29c dramatically increases the metastasic potential of 95C low-metastatic cells. Finally, we compared the differences in metastasic tendency in relation to whether miR-29c was activated. Statistic results in Table 1 confirm that miR-29c significantly inhibits tumor metastasis *in vivo*.

**Table 1:** Frequency of metastasis in nude mice xenograft model from 95D and 95C lung cancer cells using bioluminescence imaging.

Groups	Sample size	Metastasis/total (%)
95D 29c	16	3/16 (18.8%)**
95D MiNC	15	11/15 (73.3%)
95C 29ci	17	13/17 (76.5%)*
95C IhNC	18	6/18(33.3%)

** indicates P<0.01 compared to 95D MiNC; * indicates P<0.05 compared to 95C IhNC by X^2^ test.

## Discussion

In the present study, we provide evidence that miR-29c expression in high-metastatic 95D cell lines was downregulated when compared to miR-29c expression in paired low-metastatic 95C cell lines, ectopic expression and siRNA knockdown of miR-29c confirmed it directly targeted 3′-UTR of integrin β1 and MMP2 mRNA and downregulated integrin β1 and MMP2 protein expression to suppress lung cancer cell adhesion to ECM and metastasis *in vitro* and *in vivo*.

Cell adhesion to the ECM is mediated via integrins, the principle cell surface adhesion receptors. Integrins transduce intracellular signals across the plasma membrane, thereby regulating the proliferation, migration and metastasis of cancer cells [16]. Integrin β1 facilitates cancer cell proliferation, adhesion, migration and metastasis [17]–[20]. Our study showed that expression of integrin β1 protein in 95D was higher than in 95C (Figure 5 C and D). MMPs are also thought to play a major role in cell behaviors such as cell proliferation, adhesion, migration, invasion, apoptosis, and host defense [21]. MMP2 is a 72-kDa type IV collagenase, also called gelatinase A. It is frequently mentioned in relation to tumor progression, especially in relation to tumor metastasis because it spreads cancer cells from the primary location to the stroma by degrading the extracellular matrix, including the basement membrane [22]. Recent research has found many other roles that MMP2 plays in tumor progression: MMP2 is linked to EGFR and integrin signaling, which lead to cell migration, and the switch to an angiogenic phenotype in an animal model (Epithelial–Mesenchymal Transition). Silencing of MMP2 causes cancer cell apoptosis by up-regulating Fas/Fas-L and FADD [23]–[26]. Therefore, through various functions, MMP2 is closely tied to lung cancer progression, including angiogenesis and migration [27]–[29].We also found MMP2 protein expression in high-metastatic 95D was more than that of in low-metastatic 95C (Figure 5 A and B).

MicroRNAs have been shown to play an indispensible role in tumor metastasis and invasion in many types of cancer, including lung cancer [3], [9], [30]. A number of recent studies have indicated that some miRNAs are directly involved in tumor cell proliferation, invasion and metastasis [5]–[10], [31]–[33]. We found the differential expression of miRNAs in different cell lines and conducted a microarray analysis that showed several differential regulations of specific miRNAs in the 95C and 95D cell lines (Table S1). We confirmed miR-29c in high-metastatic 95D was less than that of in low-metastatic 95C by qRT-PCR (Figure 1A). Previous study found that miR-29c down-regulated protein expression of several extracellular matrix components including multiple collagens and laminin γ1 in nasopharyngeal carcinoma, and miR-29c may also directly target DNMT3A and DNMT3B in lung cancer (Fabbri, et al., 2007; Sengupta, et al., 2008). Recently, more reports have shown that miR-29 family regulated tumorigenesis and tumor progression by targeting DNMT3A, DNMT3B, TIAM1, cyclin E, MMP2, ID1, SPARC and COL3A1. These studies indicate miR-29 family can regulate different target genes in various cancer [12], [13], [32], [34]–[37].

In this study, we performed gain-of-function in 95D cells and loss-of-function in 95Ccells of miR-29c. Our data demonstrate that miR-29c inhibits *in vitro* proliferation (Figure 1B and C), adhesion to ECM (Figure 2), invasion and migration (Figure 3), and *in vivo* suppresses lung cancer distant metastasis in a nude mouse xenograft model (Figure 6). Furthermore, the dual-luciferase reporter assay demonstrated that miR-29c inhibits the expression of luciferase gene containing the 3′-UTR of integrin β1 and MMP 2 mRNA(Figure 4). Western blotting indicated that miR-29c downregulated the endogenous protein expression of integrin β1 and MMP2 (Figure 5A–D). Gelatin zymography analysis further confirmed that miR-29c decreases MMP2 enzyme activity (Figure 5 E and F). Interestingly, in gelatin zymography, MMP9 enzyme activity was also inhibited by miR-29c mimics (Figure 5 E and G). However, further investigation on whether miR-29c regulates MMP9 enzyme activity is needed.

At present, many selective inhibitor drugs, such as Gefitinib (epidermal growth factor receptor inhibitor) are widely used in clinical settinga as first- or second-line treatments. However, when Gefitinib is used on lung cancer cells, integrin β1 and MMP2 function are up-regulated and leads to Gefitinib resistance [38], [39]. A new chemotherapy agent that can deliver miR-29c and target integrin β1 and MMP2 may solve this problem because of the close relationship between MMP2 and miR-29c in tumor metastasis.

In conclusion, our studies demonstrate that miR-29c expression is down-regulated in high- metastasis 95D lung cancer cells, and that miR-29c plays a strong inhibitory role in tumor cell adhesion and metastasis. We provide important evidence that that miR-29c can suppress lung cancer cell adhesion to ECM and metastasis by targetting the 3′-UTR of integrin β1 and MMP2 mRNA to down-regulate their protein expression. Our data suggest that miR-29c provides a new potential target for lung cancer treatment.

## Supporting Information

Table S1 **Differential expression of miRNAs between high- (95D) and low-metastatic (95C) lung cancer cells.**
(DOCX)
