# Supplementary material for: miRNA-29c Suppresses Lung Cancer Cell Adhesion to Extracellular Matrix and Metastasis by Targeting Integrin β1 and Matrix Metalloproteinase2 (MMP2)
Source: PLoS One. 2013 Aug 6;8(8):e70192. doi: 10.1371/journal.pone.0070192 (PMC3735565; doi:10.1371/journal.pone.0070192)
Supplement: Table S1 — Differential expression of miRNAs between high- (95D) and low-metastatic (95C) lung cancer cells. (DOCX) [file pone.0070192.s001.docx]

Table S1 Differential expression of miRNAs between high- (95D) and low-metastatic (95C) lung cancer cells (differentially expressed transcripts with p value < 0.01).

| **No.** | **Probe_ID** | **95Ccells**  **Signal** | **95D cell Signal** | **log2 (95D / 95C)** |
| --- | --- | --- | --- | --- |
| **1** | **hsa-miR-708** | **37.68** | **251.86** | **2.68** |
| **2** | **hsa-miR-663** | **7,018.02** | **1,519.56** | **-2.24** |
| **3** | **hsa-miR-363** | **394.97** | **97.49** | **-1.95** |
| **4** | **hsa-miR-149*** | **3,853.83** | **1,079.22** | **-1.84** |
| **5** | **hsa-miR-29c** | **6,263.49** | **1,675.99** | **-1.72** |
| **6** | **hsa-miR-98** | **1,107.65** | **3,436.77** | **1.63** |
| **7** | **hsa-miR-365** | **727.89** | **2,192.59** | **1.49** |
| **8** | **hsa-miR-923** | **33,650.48** | **12,034.66** | **-1.47** |
| **9** | **hsa-miR-10b** | **2,251.24** | **6,618.80** | **1.44** |
| **10** | **hsa-miR-638** | **34,128.58** | **12,092.45** | **-1.38** |
| **11** | **hsa-miR-483-5p** | **761.22** | **272.51** | **-1.35** |
| **12** | **hsa-miR-29a** | **35,554.22** | **14,546.10** | **-1.20** |
| **13** | **hsa-miR-212** | **697.26** | **317.62** | **-1.19** |
| **14** | **hsa-miR-132** | **6,277.88** | **2,816.82** | **-1.13** |
| **15** | **hsa-miR-10a** | **6,012.25** | **13,878.99** | **1.11** |
| **16** | **hsa-miR-29b-1*** | **1,198.46** | **557.88** | **-1.07** |
| **17** | **hsa-miR-9*** | **995.73** | **1,939.95** | **1.05** |
| **18** | **hsa-miR-137** | **253.93** | **535.97** | **1.05** |
| **19** | **hsa-miR-9** | **4,229.31** | **8,191.67** | **1.01** |
| **20** | **hsa-let-7b** | **7,030.84** | **12,505.60** | **0.94** |
| **21** | **hsa-miR-181b** | **2,671.89** | **5,081.53** | **0.89** |
| **22** | **hsa-miR-148b** | **521.93** | **922.85** | **0.88** |
| **23** | **hsa-miR-19b** | **4,232.54** | **2,386.25** | **-0.83** |
| **24** | **hsa-miR-454** | **2,204.11** | **3,764.76** | **0.72** |
| **25** | **hsa-miR-181d** | **1,085.42** | **1,949.31** | **0.72** |
| **26** | **hsa-miR-194** | **1,064.53** | **660.00** | **-0.69** |
| **27** | **hsa-miR-15a** | **1,632.48** | **2,575.44** | **0.66** |
| **28** | **hsa-miR-26b** | **6,772.15** | **10,404.99** | **0.64** |
| **29** | **hsa-let-7g** | **11,990.13** | **18,882.27** | **0.64** |
| **30** | **hsa-miR-382** | **1,327.35** | **2,049.51** | **0.63** |
| **31** | **hsa-miR-374b** | **1,105.04** | **1,661.29** | **0.62** |
| **32** | **hsa-miR-7** | **4,827.50** | **7,427.96** | **0.60** |
| **33** | **hsa-miR-425** | **5,955.86** | **3,990.08** | **-0.57** |
| **34** | **hsa-let-7d** | **22,129.74** | **30,942.92** | **0.55** |
| **35** | **hsa-let-7f** | **23,615.61** | **33,267.90** | **0.54** |
| **36** | **hsa-miR-30a** | **1,690.16** | **1,159.35** | **-0.54** |
| **37** | **hsa-let-7e** | **18,944.07** | **26,335.43** | **0.54** |
| **38** | **hsa-miR-196a** | **3,481.25** | **5,032.69** | **0.53** |
| **39** | **hsa-miR-493*** | **1,266.43** | **1,801.68** | **0.53** |
| **40** | **hsa-miR-192** | **1,828.46** | **1,279.71** | **-0.50** |
| **41** | **hsa-miR-195** | **4,197.16** | **2,833.69** | **-0.49** |
| **42** | **hsa-let-7a** | **25,610.25** | **35,875.44** | **0.49** |
| **43** | **hsa-miR-181a** | **2,489.78** | **3,289.92** | **0.48** |
| **44** | **hsa-miR-432** | **2,257.01** | **3,099.78** | **0.46** |
| **45** | **hsa-let-7c** | **22,082.72** | **29,289.98** | **0.46** |
| **46** | **hsa-miR-494** | **3,180.46** | **2,094.45** | **-0.46** |
| **47** | **hsa-miR-155** | **34,813.97** | **24,134.39** | **-0.45** |
| **48** | **hsa-miR-127-3p** | **1,931.51** | **1,427.37** | **-0.44** |
| **49** | **hsa-miR-130b** | **2,392.53** | **3,174.74** | **0.43** |
| **50** | **hsa-miR-106b** | **10,654.02** | **7,945.16** | **-0.40** |
| **51** | **hsa-miR-30c** | **8,280.04** | **6,395.20** | **-0.38** |
| **52** | **hsa-miR-222** | **13,175.33** | **10,279.30** | **-0.37** |
| **53** | **hsa-miR-183** | **2,752.67** | **3,508.32** | **0.37** |
| **54** | **hsa-miR-27b** | **5,202.22** | **6,497.20** | **0.35** |
| **55** | **hsa-let-7i** | **15,377.59** | **19,266.11** | **0.33** |
| **56** | **hsa-miR-25** | **22,269.41** | **17,857.23** | **-0.32** |
| **57** | **hsa-miR-320** | **12,680.10** | **16,448.34** | **0.27** |
| **58** | **hsa-miR-16** | **26,860.05** | **23,451.77** | **-0.26** |
| **59** | **hsa-miR-31** | **13,095.63** | **10,922.66** | **-0.25** |
| **60** | **hsa-miR-21** | **47,845.95** | **39,965.91** | **-0.24** |
| **61** | **hsa-miR-221** | **14,798.43** | **12,823.80** | **-0.21** |
| **62** | **hsa-miR-26a** | **16,602.75** | **18,502.74** | **0.16** |
| **63** | **hsa-miR-23a** | **30,255.29** | **32,953.49** | **0.15** |
| **64** | **hsa-miR-15b** | **27,512.47** | **30,568.90** | **0.14** |
